# Mitochondrial function is involved in regulation of cholesterol efflux to apolipoprotein (apo)A-I from murine RAW 264.7 macrophages

**DOI:** 10.1186/1476-511X-11-169

**Published:** 2012-12-10

**Authors:** Anne Marie Allen, Annette Graham

**Affiliations:** 1 Department of Life Sciences, School of Health and Life Sciences and the Diabetes Research Group, Institute for Applied Health Research, Glasgow Caledonian University, Cowcaddens Road, Glasgow, G4 0BA, UK

**Keywords:** Cholesterol efflux, Cholesterol esterification, Liver X Receptor, ATP binding cassette transporter A1, Apolipoprotein A-I, High density lipoprotein

## Abstract

**Background:**

Mitochondrial DNA damage, increased production of reactive oxygen species and progressive respiratory chain dysfunction, together with increased deposition of cholesterol and cholesteryl esters, are hallmarks of atherosclerosis. This study investigated the role of mitochondrial function in regulation of macrophage cholesterol efflux to apolipoprotein A-I, by the addition of established pharmacological modulators of mitochondrial function.

**Methods:**

Murine RAW 264.7 macrophages were treated with a range of concentrations of resveratrol, antimycin, dinitrophenol, nigericin and oligomycin, and changes in viability, cytotoxicity, membrane potential and ATP, compared with efflux of [^3^H]cholesterol to apolipoprotein (apo) A-I. The effect of oligomycin treatment on expression of genes implicated in macrophage cholesterol homeostasis were determined by quantitative polymerase chain reaction, and immunoblotting, relative to the housekeeping enzyme, *Gapdh*, and combined with studies of this molecule on cholesterol esterification, *de novo* lipid biosynthesis, and induction of apoptosis. Significant differences were determined using analysis of variance, and Dunnett’s or Bonferroni post *t*-tests, as appropriate.

**Results:**

The positive control, resveratrol (24 h), significantly enhanced cholesterol efflux to apoA-I at concentrations ≥30 μM. By contrast, cholesterol efflux to apoA-I was significantly inhibited by nigericin (45%; *p*<0.01) and oligomycin (55%; *p*<0.01), under conditions (10 μM, 3 h) which did not induce cellular toxicity or deplete total cellular ATP content. Levels of ATP binding cassette transporter A1 (ABCA1) protein were repressed by oligomycin under optimal efflux conditions, despite paradoxical increases in *Abca1* mRNA. Oligomycin treatment did not affect cholesterol biosynthesis, but significantly inhibited cholesterol esterification following exposure to acetylated LDL, and induced apoptosis at ≥30 μM. Finally, oligomycin induced the expression of genes implicated in both cholesterol efflux (*Abca1*, *Abcg4*, *Stard1*) and cholesterol biosynthesis (*Hmgr*, *Mvk*, *Scap*, *Srebf2*), indicating profound dysregulation of cholesterol homeostasis.

**Conclusions:**

Acute loss of mitochondrial function, and in particular Δψ_m_, reduces cholesterol efflux to apoA-I and dysregulates macrophage cholesterol homeostasis mechanisms. Bioavailable antioxidants, targeted to mitochondria and capable of sustaining effective mitochondrial function, may therefore prove effective in maintenance of arterial health.

## Background

Atherosclerosis is associated with increased production of reactive oxygen species (ROS) in mitochondria, accumulation of mitochondrial DNA damage, and progressive respiratory chain dysfunction [1-4]. In turn, mitochondrial dysfunction is induced by hypercholesterolemia, hyperglycemia, hypertriglyceridemia, and even the process of aging. Increased oxidative stress within the artery wall modifies low-density lipoprotein (LDL) to a form recognised by macrophage scavenger receptors, resulting in the unregulated uptake of excess cholesterol and cholesteryl esters within macrophage ‘foam cells’, a hallmark of early and developing atheroma [1-5].

Regression and stabilisation of atherosclerotic lesions requires the efficient removal of cholesterol from macrophage ‘foam cells’ to apolipoprotein (apo)A-I or apoE, allowing nascent high density lipoprotein (HDL) particles to enter and mature within the reverse cholesterol transport (RCT) pathway in the bloodstream, which delivers excess cholesterol to the liver for excretion as bile and bile acids [6]. Recently, we demonstrated that mitochondrial cholesterol transport is a key step controlling macrophage cholesterol efflux, the first step in the RCT pathway [7]. Notably, overexpression of steroidogenic acute regulatory protein (StAR; StarD1) in macrophages increases delivery of cholesterol to sterol 27-hydroxylase (CYP27A1), located on the inner mitochondrial membrane [7]. Sterol 27-hydroxylase generates oxysterol ligands for Liver X Receptors (LXRα/β); nuclear transcription factors which act as master regulators of genes encoding proteins involved in the cholesterol efflux pathway [8]. These include ATP binding cassette (ABC) transporters, such as ABCA1 and ABCG1/ABCG4, which work in concert to transfer cholesterol across the plasma membrane to apoA-I and HDL, respectively [9,10].

High levels of sterol 27-hydroxylase are found to co-localise with macrophages in human atherosclerotic lesions [11], and 27-hydroxycholesterol is a major oxysterol in human atheroma [12]. The importance of CYP27A1 in macrophage cholesterol homeostasis is highlighted by the rare genetic disorder, cerebrotendinous xanthomasis, which is characterized by increased risk of premature atherosclerosis, despite normal concentrations of cholesterol in the bloodstream [13]. Importantly, the rate-limiting step controlling the activity of sterol 27-hydroxylase is the supply of cholesterol to the enzyme, which can be mediated by the interaction of StAR with a protein complex located at contact sites between outer and inner mitochondrial membranes [7,14-16].

These findings, together with studies on mitochondrial cholesterol transport in steroidogenic tissues [16-19] led directly to our original hypothesis: that mitochondrial function be intrinsically linked to efficiency of macrophage cholesterol efflux, with consequences for other cholesterol homeostasis mechanisms. Certainly, transfer of cholesterol from the outer to the inner mitochondrial membrane requires energised, polarised and actively respiring mitochondria [16-20], while compounds such as resveratrol (3, 4’, -5-trihydroxy-*trans*-stilbene), which protect mitochondrial function by inducing mitochondrial biogenesis [21] and reducing oxidative stress [22], are also reported to increase macrophage cholesterol efflux [22-24]. However, resveratrol is also reported to induce the expression of LXRα [23] and to impact positively on cholesterol flux in human cells via PPAR γ and adenosine [24], suggesting pleiotropic modes of action for this molecule.

The aim of this study was to use established and specific modulators of mitochondrial function (antimycin, dinitrophenol, nigericin and oligomycin) to test whether a causal link exists between loss of mitochondrial function and impaired macrophage cholesterol efflux. Changes in cell viability and cytotoxicity, membrane potential and total ATP levels were compared with the ability of macrophages to efflux cholesterol to apoA-I; resveratrol was included as a putative positive control in these experiments, as this molecule is known to exert hormetic and cell-specific effects [25]. The results have implications for diseases associated with impaired mitochondrial function, including ageing, atherosclerosis, Alzheimer’s disease and diabetes.

## Results

**Figure 1:**
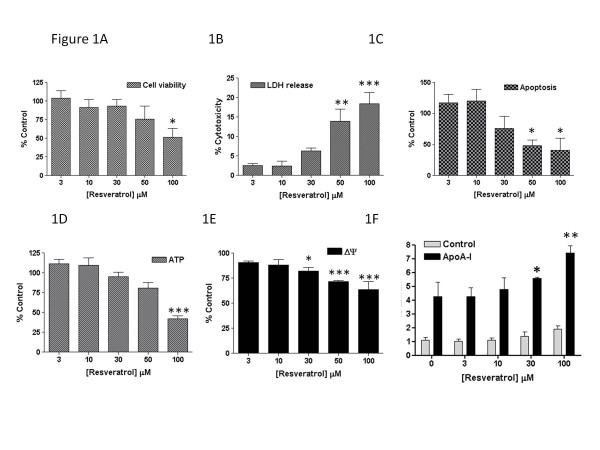
**The effect of resveratrol, used at the concentrations indicated for 24 h on A) cell viability, B) cytotoxicity, C) apoptosis, D) total cellular ATP levels, E) mitochondrial membrane potential, and F) efflux of [**^**3**^**H]cholesterol to apoA-I (20**μ**g ml**^**-1**^**), as described in the Methods.** Values are the mean±SEM of between three to four independent experiments; *p<0.05; **p<0.01 and ***p<0.001 compared with control macrophages.

The first part of this study set out to confirm the reported positive impact of resveratrol on cholesterol efflux, in particular under conditions of demonstrably unimpaired (or improved) mitochondrial function and sustained cellular viability [21-24]. The impact of resveratrol (0-100 μM; 24 h) on the conversion of MTT to formazan is shown in Figure 1A, with a significant decline in cell viability noted at 100 μM. Concentrations of 50 μM (*p*<0.01) and 100 μM (*p*<0.001) were associated with significantly increased release of cytosolic LDH into the medium (Figure 1B), and decreased Caspase 3/7 activity (*p*<0.05) (Figure 1C), suggesting cellular necrosis, rather than induction of apoptosis. Levels of ATP showed a similar pattern of decline, with a significant 58% reduction (*p*<0.001) noted at 100 μM resveratrol (Figure 1D). Interestingly, despite the proposed functions of resveratrol [21-24], this compound impacted negatively on membrane potential, with minor but reproducible losses noted at concentrations ≥30 μM (Figure 1E), concentrations used previously to demonstrate cardioprotective properties for this compound [23]. As previously described [22], resveratrol significantly increased the efflux of ^3^H]cholesterol (Figure 1F) to apoA-I (20 μg ml^-1^), but only at the limit of this concentration range (30 μM) and not at lower concentrations, when tested under optimal efflux conditions (0.3 mM dibutyryl cAMP [20]). These findings therefore provide only limited evidence for a positive impact of this ‘nutriceutical’ on macrophage cholesterol efflux, suggesting that other properties of this compound may be more important in conferring cardioprotection [21-24].

**Figure 2:**
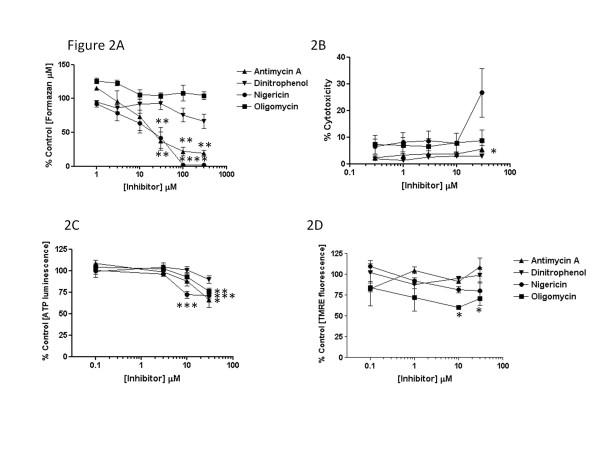
**The effects of mitochondrial inhibitors, antimycin, dinitrophenol, nigericin and oligomycin, used at the concentrations indicated for 3 h on A) cell viability, B) cytotoxicity, C) total cellular ATP levels and D) mitochondrial membrane potential, as described in the Methods.** Values are the mean±SEM of between three to four independent experiments; *p<0.05; **p<0.01 and ***p<0.001 compared with control macrophages.

The second part of this study therefore sought evidence that loss of mitochondrial function diminishes the atheroprotective cholesterol efflux pathway, again under conditions *without* overt loss of cell viability, release of cytosolic LDH or loss of total cellular ATP; reductions in mitochondrial membrane potential were, however, predicted for some compounds by previous studies [16-19,26], and formed part of the hypothesis under test. Mitochondrial inhibitors, nigericin and antimycin, decreased cellular viability (Figure 2A) and/or tended to increase release of cytosolic LDH into the medium (Figure 2B) at concentrations ≥ 30 μM after 3 h, while dinitrophenol or oligomycin had no significant impact on either parameter during this period. By contrast, more prolonged incubation (24 h) with these inhibitors caused substantive loss of cell viability, with estimated LD_50_ values of 1 μM (antimycin), 75 μM (dinitrophenol), 1 μM (nigericin) and 8 μM (oligomycin), as judged by MTT conversion to formazan (*data not shown*). During a 3 h incubation, intracellular levels of ATP were depleted by oligomycin (30 μM) and nigericin (≥10 μM) by 24% (*p*<0.01) and 28% (*p*<0.001), respectively, but not by the other drugs tested (Figure 2C), while mitochondrial membrane potential declined significantly by 39% (*p*<0.05) only in the presence of oligomycin (≥10μM).

**Figure 3:**
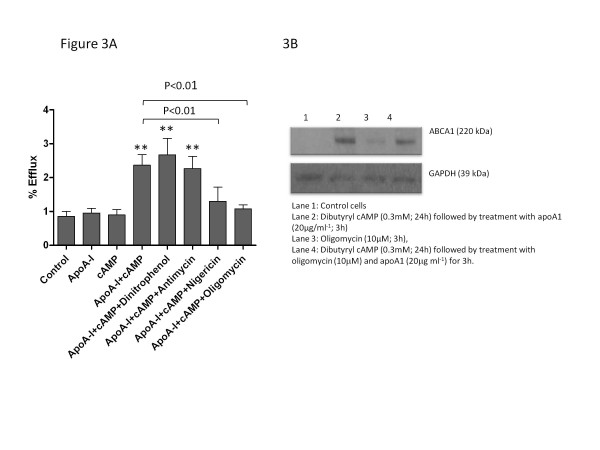
**The effects of mitochondrial inhibitors, antimycin, dinitrophenol, nigericin and oligomycin, each used at 10μM for 3 h, on efflux of [**^**3**^**H]cholesterol to apoA-I (20** **μg ml**^**-1**^**) is shown in Figure**3A. Where indicated, macrophages were preincubated for 24 h in the presence of dibutyryl cAMP (0.3 mM) to induce expression of ABCA1 and cholesterol efflux [25]. Values are the mean ± SEM of between four to six independent experiments; **p<0.01 compared with control macrophages; other significant changes are indicated on the figure. Expression of ABCA1 protein under the conditions noted is shown in Figure 3B; values are shown relative to the housekeeping gene, GAPDH, and are representative of three independent experiments.

When tested at concentrations which did not affect cellular viability (10 μM; 3 h), only nigericin and oligomycin inhibited efflux of [^3^H] cholesterol to apoA-I (Figure 3A) by 45% (*p*<0.01) and 55% (*p*<0.01), respectively, following preincubation with dibutyryl cAMP (0.3 mM). By contrast, neither antimycin nor dinitrophenol significantly changed cholesterol efflux to apoA-I. While macrophage levels of ABCA1 protein were modestly increased following oligomycin treatment, levels of this protein were repressed by oligomycin (34±17%; *n=3*; *p*<0.05) in macrophages incubated under optimal efflux conditions (0.3 mM dibutyryl cAMP and 20μg ml^-1^ apoA-I), and by comparison with GADPH (Figure 3B).

**Figure 4:**
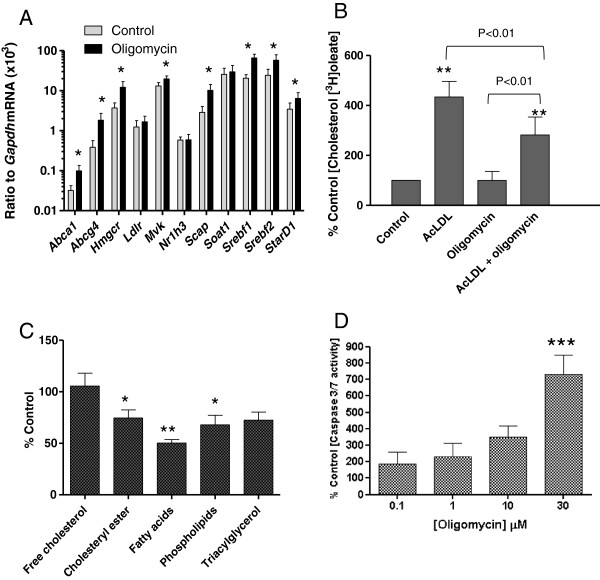
**The effect of oligomycin (10** **μM; 3 h) on A) fold-change of genes involved in cholesterol metabolism and lipoprotein signalling in RAW 264.7 macrophages, compared with control cells, as judged by Q-PCR analysis of genes implicated in the cholesterol efflux, biosynthesis and esterification pathways; B) incorporation of [**^**3**^**H]oleate (10** **μM) into the cholesteryl ester pool, measured in the presence of absence of acetylated LDL (50** **μg ml**^**-1**^**; 3 h); C) incorporation of [**^**14**^**C]acetate into free cholesterol, cholesteryl ester, fatty acids, phospholipids and triacylglycerol (3 h) and D) induction of apoptosis in RAW 264.7 murine macrophages.** Values are the mean±SEM of between three to six independent experiments. *p<0.05, **p<0.01 and ***p<0.001 compared with control macrophages.

Oligomycin treatment induced gene expression of *Abca1* and *Abcg4* mRNA, together with genes encoding other enzymes and transcription factors involved in cholesterol metabolism, including *Hmgcr*, *Mvk*, *Scap1*, *Srebf1*, *Srebf2* and *Stard1* (Figure 4A). Further, when macrophages were incubated in the presence of acetylated LDL (50 μg ml^-1^) to induce ‘foam cell’ formation, oligomycin (10 μM; 3 h) inhibited acyl CoA: cholesterol acyltransferase (ACAT1) activity by 35% (*p*<0.01), as judged by incorporation of [^3^H]oleate (10 μM) into the cholesteryl ester pool (Figure 4B). Equally, under basal conditions, oligomycin (10 μM; 3 h) significantly decreased incorporation of [^14^C]acetate into cholesteryl ester, fatty acid and phospholipid pools, while biosynthesis of free [^14^C]cholesterol remained unaffected (Figure 4C). Finally, increasing concentrations of oligomycin were associated with a trend towards increasing macrophage apoptosis (Figure 4D), which proved significant at 30 μM.

## Discussion

Loss of mitochondrial function, a key element of atherogenesis, is caused by an increase in reactive oxygen species, resulting in accumulation of mitochondrial DNA damage, and respiratory chain deficiencies, apoptosis and cell death, favouring plaque formation and instability [1-5]. This study argues that dysregulation of macrophage cholesterol homeostasis should also join this list, apparently driven by the specific loss of macrophage cholesterol efflux to apoA-I.

Notably, resveratrol, a natural phytoalexin antioxidant found in grapes and red wine, known to induce mitochondrial biogenesis and protect against atherosclerosis in animal models [23,25], increased cholesterol efflux to apoA-I, at concentrations around 20-30 μM, as previously described [22,24]. However, it is evident that concentrations of resveratrol (30-100 μM) which increase cholesterol efflux (Figure 1), and indeed those reported to activate LXRα [23], are associated with loss of mitochondrial membrane potential and cell viability and induction of cytotoxicity in RAW 264.7 macrophages. Moreover, since resveratrol is characterised by high absorption but very low bioavailability, coupled with extensive and rapid metabolism to conjugates of this compound [27], it seems unlikely that monocyte-macrophages would be exposed to the concentrations of resveratrol needed to stimulate the cholesterol efflux process. For example, absorption (70%) of a dietary relevant 25 mg oral dose generated peak plasma levels of around 2 μM resveratrol metabolites, with only trace amounts of unchanged resveratrol detected in the plasma (<5 ng ml^-1^) [27]. However, it may be possible that the more potent antioxidant function of resveratrol (≤5 μM) [22], or the ability of this compound to amplify SIRT-1 dependent biogenesis of mitochondria (≤10 μM) [21], might help to sustain mitochondrial function in vivo, particularly if local depots of resveratrol accumulate within arterial cells. If so, macrophages may have to be oxidatively challenged, or subject to chronic incubation with resveratrol, for the protective impact of this compound on the cholesterol efflux pathway to become more (patho)physiologically relevant.

By contrast, inhibitors of differing aspects of mitochondrial function (ΔpH, Δψ_m_ complex III or complex V) exerted convincing, if selective, effects on the cholesterol efflux pathway over the acute time period tested here. Notably, the uncoupling agent, dinitrophenol, and complex III inhibitor, antimycin, had negligible impact on efflux to apoA-I, while nigericin and oligomycin both reduced efflux to apoA-I. While nigericin (10 μM) decreased total cellular ATP levels, which could impact on the activity of ABC transporters, ABCA1 and ABCG1/G4, oligomycin did not alter cell viability or total ATP levels, but did significantly reduce Δψ_m_, unlike the other inhibitors tested. In turn, loss of Δψ_m_ due to oligomycin was associated with inhibition of cholesterol esterification and a trend towards induction of apoptosis, together with increased expression of an array of genes involved in cholesterol homestasis, arguing a profound dysregulation of cholesterol homeostasis as sequelae of acute loss of mitochondrial function. Importantly, the coordinated response which should link impaired cholesterol efflux with SREBP-2 repression of cholesterol biosynthesis and LDL uptake, and with increased cholesterol esterification, seems to be lost.

While this is the first study to focus on the importance of mitochondria in regulation of macrophage cholesterol efflux, related studies have been performed in steroidogenic cells [16–19; 26]. As previously introduced, the rate-limiting step in the generation of steroid hormones, is the transfer of cholesterol into mitochondria, to the CYP11A1 protein which resides on the inner mitochondrial membrane. Dissipation of Δψ_m_with CCCP, inhibition of electron transport using antimycin A, disruption of pH using nigericin, and inhibition of F_0_/F_1_ ATP synthase using oligomycin, all inhibited progesterone synthesis in Leydig cells, indicating that altered mitochondrial function regulates steroid biosynthesis [16–19; 26]. However, in RAW 264.7 macrophages, only nigericin and oligomycin regulated macrophage cholesterol efflux to apoA-I, and at concentrations 10-fold higher than those required to inhibit steroidogenesis [16-19,26]. It is noteworthy that cellular ATP content was more sensitive to depletion by mitochondrial disruption in Leydig cells than in RAW 264.7 macrophages, as only oligomycin (≥30 μM) and nigericin (≥10 μM) reduced cellular ATP levels over the same time scale in the latter; equally, Δψ_m_ was markedly reduced by antimycin (1 μM) and oligomycin (1 μM) in Leydig cells, while only oligomycin (≥10 μM) affected this parameter in the current study. This may explain the apparent selectivity for nigericin and oligomycin in repression of cholesterol efflux to apoA-I; certainly, loss of either Δψ_m_ or cellular ATP seems sufficient to negatively affect macrophage cholesterol efflux when cell viability is sustained (Figures 2 and 3).

Oligomycin treatment, by limiting cholesterol efflux (Figure 2A) and reducing cholesterol esterification (Figure 4B), without impacting on cholesterol biosynthesis (Figure 4C), should therefore lead to accumulation of sterol at the endoplasmic reticulum (ER). This should trigger a protective cholesterol homeostasis response, sequestering SREBPs at the ER, and providing oxysterol ligands for Liver X Receptors (LXRs) complexed with retinoid X receptors (RXR) at the LXR response element within genes involved in the cholesterol efflux pathway [8]. Sterol-dependent sequestration of SREBPs at the ER prevents proteolytic processing of this transcription factor to the nuclear SREBP fragment capable of inducing the transactivation of genes involved in cholesterol biosynthesis and uptake. However, instead of these correctly orchestrated events, treatment with oligomycin resulted in upregulation of genes involved in both cholesterol biosynthesis (*Hmgr, Mvk, Scap, Srebf1, Srebf2*) and efflux (*Abca1, Abcg4, StarD1*) pathways, arguing a profound dysregulation of this response. The mechanism involved was not investigated here, but it is possible to speculate that loss of mitochondrial production of oxysterol LXR ligands [7] may release SREBP2 from the ER, explaining the increased expression of genes involved in increasing cholesterol biosynthesis. This could then result in increased production of 24(S),25-epoxycholesterol via the cholesterol biosynthetic pathway [28], facilitating LXR activation and induction of genes involved in the efflux process.

One further outcome predicted by the macrophage lipid phenotype observed following oligomycin treatment is toxic overaccumulation of cholesterol at the ER, which can trigger oxidative stress and proteasomal degradation of existing ABCA1 protein [29] and ultimately trigger apoptosis, observed at higher concentrations of oligomycin (Figure 4D). While it is clear that oligomycin induction of *Abca1* mRNA may explain the modest increase in ABCA1 protein under basal conditions, a clear reduction in ABCA1 is noted when macrophages are treated with this inhibitor under optimal efflux conditions (Figure 3B), which agrees well with the observed loss of ^3^H]cholesterol efflux to apoA-I under this condition (Figure 3A). The dissociation between expression of ABCA1 mRNA and protein in oligomycin-treated macrophages under this condition reflects that observed in carotid atherosclerotic lesions, where elevated *Abca1* mRNA is associated with reduced expression of ABCA1 protein [30].

## Conclusions

Loss of mitochondrial function impacts negatively on macrophage cholesterol homeostasis, reducing efflux to apoA-I and impairing cholesterol esterification, events which may trigger accumulation of toxic free cholesterol and induction of apoptosis within arterial macrophages, contributing to lesion development. Small drug molecules, specifically targeted to accumulate within the mitochondria [31] and capable of sustaining Δψ_m_ might, therefore, not only reduce arterial oxidative stress, but also improve cholesterol homeostasis and efflux from macrophage ‘foam’ cells, regressing and stabilising atherosclerotic plaque.

## Methods

### Materials

Murine RAW 264.7 macrophages were purchased from the European Collection of Cell Cultures (ECACC; Health Protection Agency Culture Collection, Porton Down, UK). Tissue culture reagents were purchased from Lonza (Wokingham, UK); other sources include FuGene6 transfection reagent (Roche), NuPAGE gels and buffers (InVitrogen), antibodies (AbCAM, Cambridge, UK), primers and probes (Eurogentec, Belgium). Athens Research (USA) provided apoA-I and low density lipoprotein (LDL); LDL was acetylated according to Brown et al. [32]. Radiolabels (^3^H]cholesterol and ^14^C]acetic acid) were provided by ICN Biologicals; all other chemicals were provided by Sigma-Aldrich (Poole, Dorset, UK).

### Cell Culture

Murine RAW 264.7 macrophages were sustained using a split ratio of 1:4, in Dulbecco’s Modified Eagles Medium (DMEM) supplemented with foetal bovine serum (FBS, 10%, v/v), L-glutamine (200 mM) and penicillin/streptomycin (50 μg ml^-1^; 50 U ml^-1^, respectively). For experiments, cells were plated onto 12-well tissue culture dishes at an initial density of 0.5x10^6^ cells well^-1^, under serum-free conditions, except where stated. Compounds with an established role in modulation of mitochondrial function (resveratrol, antimycin, dinitrophenol, nigericin and oligomycin) were added using DMSO (<0.01%) as vehicle, at the concentrations and time periods indicated in the Legends to Figures.

### Macrophage lipid analysis and cholesterol efflux

Incorporation of ^14^C]acetic acid (1 μCi ml^-1^) into fatty acid, phospholipid, cholesterol, cholesteryl ester and triacylglycerol pools were measured after incubation for 3 h in the presence of oligomycin (10 μM), as previously described [7,33]. Esterification of cholesterol, in the presence of acetylated LDL (50 μg ml^-1^) was monitored by flux of ^3^H]oleic acid (10 μM; 1 μCi ml^-1^) into cholesteryl ^3^H]oleate, as previously [33]. Macrophage lipids were extracted using hexane:isopropanol (3:2, v/v) and dried under nitrogen, before separation by t.l.c. using chloroform, methanol and water (60:30:5, by vol.) as the first mobile phase, and hexane, diethyl ether and acetic acid (80:20:1.5, by vol.) in the second phase of development; lipids were identified by co-migration with authentic standards [7,33].

Macrophage efflux of ^3^H]cholesterol to apoA-I and HDL were determined as previously described [7,33]. Efflux from radiolabelled macrophages, was initiated by addition of serum-free DMEM containing human apoA-I (20 μg ml^-1^) or HDL (20 μg ml^-1^) for 3 h; preincubation (24 h) with dibutyryl cAMP (0.3 mM) is required in this cell line to induce ABCA1, as previously described [20]. Results are expressed as % cholesterol efflux, calculated as [dpm_(media)_/dpm_(media+cells)_ x 100%.

### Analysis of mRNA and protein expression

Total RNA was isolated (TriZol) from macrophages, and reverse transcribed in cDNA (BioLine) prior to measurement of cellular levels of *Abca1*, *Ldlr*, *Mvk*, *Nr1h3*, *Scap*, *Soat1*, *Srebf1* and *Srebf2* by quantitative polymerase chain reaction (Q-PCR), relative to *Gapdh*, using primers and fluorescent (FAM/TAMRA) probe sequences, and protocols cited in [7]. Levels of *Abcg4* were determined using forward (5’-GCGCCTGGCCATTGC-3’), reverse (5’-GACCGCTGGTAGGCTCATCA-3’) primer and probe (5’-CTGGTCAACAACCCGCCTGTCATGT-3’) sequences (100nM).

Macrophage cell lysates were prepared in RIPA buffer (25 mM Tris HCl pH7.6, 150 mM NaCl, 1% (v/v) NP-40, 1% (w/v) sodium deoxycholate, 0.1% (w/v) sodium dodecyl sulphate) supplemented with Complete™ protease inhibitor cocktail (Roche), and proteins (50 μg lane^-1^) separated by SDS PAGE (NuPAGE; 10% gels), transferred to a PDVF membrane and probed using anti-ABCA1 (1:1000) and anti-GAPDH (1:2000) rabbit polyclonal antibodies, and detection achieved using secondary antibodies coupled to the ECL detection system [7,33].

### Assessment of macrophage viability, apoptosis and mitochondrial function

Cell viability was assessed by conversion of methyl thiazolyl blue tetrazolium bromide to formazan, via succinate: ubiquinol oxidoreductase (Complex II), as previously [33], and by release of lactate dehydrogenase into the medium (LDH Cytotoxicity Assay Kit, Cambridge Biosciences). Membrane potential was assessed by tetramethylrhodamine fluorescence [17], total cellular ATP using the luciferase Celltiter Glo-Bioluminescent reagent supplied by Promega, and apoptosis by monitoring Caspase 3/7 activity (Promega).

All values indicate mean±S.E.M., with numbers of independent experiments denoted by *n*. Significant (*p*<0.05) differences were determined using analysis of variance and Dunnett’s, or Bonferroni post *t*-tests, as appropriate.

## Competing interests

The authors declare that they have no competing interests.

## Author’s contributions

AMA carried out the experimental work on this study, which was jointly designed by AMA and AG. The manuscript was prepared by AG. Both authors have read and approved the final manuscript.
